# Asphaltene-Stabilized Polyisobutylene Pressure-Sensitive Adhesives for Ultraviolet Protection and Surface Bonding

**DOI:** 10.3390/ma16031209

**Published:** 2023-01-31

**Authors:** Viktoria Y. Melekhina, Anna V. Kostyuk, Nina M. Smirnova, Sergey O. Ilyin

**Affiliations:** A.V. Topchiev Institute of Petrochemical Synthesis, Russian Academy of Sciences, 29 Leninsky Prospect, 119991 Moscow, Russia

**Keywords:** UV protection, pressure-sensitive adhesives, asphaltenes, glass bonding, rheology, polyisobutylene

## Abstract

The usual way to protect indoor areas from solar UV radiation is to use UV-absorbing materials, which are applied as a thin film on the surface of the windowpane. Asphaltenes are useless wastes from crude oil refining that absorb UV radiation well, which gave the idea of their use in protective coatings. Pressure-sensitive adhesives based on polyisobutylene containing from 5 to 30 wt% of asphaltenes were obtained. Deterioration of the adhesive properties with the introduction of 5–20 wt% of asphaltenes was shown by adhesion tests, which can be associated with the plasticization of the polymer matrix. At the same time, the use of 30 wt% of asphaltenes leads to the polymer matrix reinforcement with the restoration of adhesive properties to the original level or even slightly higher. The rheological study of adhesives at 25 °C and 120 °C showed the structural network formation by asphaltenes at a content of 30 wt%, explaining the increase in adhesion performance. According to microscopy, asphaltenes are flat brown glass shards in a polymer matrix. They absorb electromagnetic radiation, predominantly in the UV range, while maintaining relative translucency in the visible range. This makes it possible to obtain thin films from the asphaltene-filled adhesive for bonding glass sheets to produce UV-blocked and tinted windowpanes.

## 1. Introduction

Ultraviolet (UV) light causes various problems in living organisms, such as itching, burning, erythema, premature aging, skin cancer, and reduced immunity [1,2,3,4]. The most essential types of UV radiation are long-wave UV-A (400–315 nm), medium-wave UV-B (315–280 nm), and short-wave UV-C (280–200 nm) [5,6]. UV-C is the highest-energy and most hazardous type, but is generally absorbed by the ozone layer in the atmosphere [7]. UV-A radiation penetrates deep into the skin and can reach the dermis, which leads to skin aging, erythema, or sunburn [8], whereas UV-B has a lower ability to penetrate the skin, but can still penetrate its epidermal layer, leading to DNA damage and skin cancer [9]. UV radiation not only affects living organisms, but also the materials surrounding them: it causes the discoloration of pigments and dyes, the yellowing and cracking of plastics, and the loss of a material’s strength properties and gloss. UV light damages both the interior materials of cars and the skin of passengers [10], poses a danger to art and archival collections in naturally-lit galleries [11], can inhibit the growth and development of crops [12], and passes through the office building windows, damaging the skin of employees.

UV protection can be implemented by incorporating UV absorbers into a photostable transparent carrier matrix for application to glass, polymer, or wood substrates. At first, Ni-containing compounds were mainly used as UV absorbers for the stabilization of low-density polyethylene films applied in agri- and horticulture [13,14]. Currently, inorganic oxides, such as CeO_2_, TiO_2_, ZnO, and SiO_2_, are more commonly used to obtain UV protective coatings [10,15,16,17,18,19]. These compounds can be used as pigments in a binder or applied as a pure oxide layer from the liquid or gaseous phase. In addition, organic UV stabilizers are widely used. Their molecules most often include a phenol group, which plays an important role in dissipating the absorbed energy. Phenolic UV absorbers are compounds containing O–H–O bridges (salicylates, 3-hydroxyflavones, 2,2′-dihydroxybenzophenones, 2-hydroxybenzophenones, and xanthones) or O–H–N bridges (2-(2-hydroxyphenyl)-1,3,5-triazines and 2-(2-hydroxyphenyl)benzotriazoles) [20,21,22]. The most important non-phenolic UV absorbers are cyanoacrylates and oxanilides [23,24], which exhibit high photochemical stability.

UV-protective polymeric films can be used as adaptable coatings for a wide variety of UV-sensitive environments. In the case of window glasses, a polymer film (UV filter) can be applied to their surface or placed between two glasses. The latter method is preferable, as it allows protection of the film from mechanical damage and removal from the glass surface. In this case, the film must have good adhesion to the glass and essentially act as an adhesive that bonds the two glass sheets together. Polymer adhesives can be of two types: hot-melts becoming sticky when heated [25,26] and pressure-sensitive adhesives (PSAs) becoming sticky when pressed [27,28]. The first adhesive is less interesting since heating requires additional energy and can lead to internal stresses when the adhesive joint is cooled due to the difference in the thermal expansion coefficients of the glued materials. This makes PSAs the preferred choice when developing UV filters for windowpanes and other kinds of glasses.

PSAs are products designed to form an adhesive bond with a substrate at low contact pressure for a short time [29,30]. PSAs have long viability, do not harden over time, and do not dry out; they are widely applied in industry, everyday life, and medicine [31]. Poly(ethylene-vinyl acetate) [32], polyurethanes [33], polyacrylates [34], polyvinylpyrrolidone [35], and some other polymers [36] are widely used as PSA polymer matrices. However, only PSAs based on polyisobutylene (PIB) are characterized by a combination of high stickiness, good oxidative and thermal stability, high availability, low price, and nontoxicity; but their problem is the propensity for irreversible distortion under the influence of small stresses—cold flow [37]. One of the options for suppressing cold flow is the use of dispersed fillers [38,39]. Their introduction makes it possible to improve the cohesive strength of PSA and increase its resistance to shear loads [40], but is accompanied by a slight decrease in stickiness and peel resistance. The most used fillers for PSA are montmorillonite [41,42], silicon dioxide [43,44], and wax [45,46], but some specialized fillers are also possible, e.g., graphene [47]. The introduction of nanosized fillers can lead to the creation of nanocomposites and the achievement of the most notable changes in the properties of final materials [48,49], and the most significant improvement in the properties occurs when applying nanofillers with anisometric particles, e.g., those that have the shape of plates [50]: montmorillonite [51,52,53,54] and graphene [55,56,57]. However, the practical use of graphene is significantly limited due to its high price, and there is one more material with flat nanosized particles, attracting the greatest attention of researchers for use in various purposes—asphaltenes [58].

Asphaltenes are part of heavy crude oil, determine its high viscosity, and remain after its deasphalting [58,59,60]. Their rational disposal is an important environmental problem, which is solved by using asphaltenes in the production of plastics, emulsions, or carbon fibers [61,62]. Asphaltenes have been used to improve epoxy plastics [63,64,65,66], polyethylene [67,68,69,70], polypropylene [71], poly(methyl methacrylate) [72], polycarbonate [73], polystyrene [74,75], and styrene copolymers [76,77,78]. Asphaltenes can act as UV absorbers [79] to prevent the aging of polymers [80], i.e., can serve as an alternative to traditional UV absorbers. On this basis, this study aims to obtain and investigate composite pressure-sensitive adhesives based on a polyisobutylene matrix filled with asphaltenes and suitable for use as a sticky translucent UV filter for glass bonding.

## 2. Materials and Methods

### 2.1. Materials

A miscible blend consisting of 10 wt% polyisobutylene BASF Oppanol B 100 (*M*_w_ = 1.1·10^6^ g/mol), 40 wt% polybutene INEOS Oligomers Indopol H-1900 (*M*_w_ = 4500 g/mol), and 50 wt% polyisobutylene BASF Oppanol B 12 (*M*_w_ = 5.1·10^4^ g/mol) was used as a base polyisobutylene pressure-sensitive adhesive (PIB) [43,46]. The mixing of three polymers with an analogous chemical structure but different molecular weight is because a polymer should have wide molecular weight distribution in order to possess the set of rheological properties necessary to exhibit the characteristics of PSA: stickiness when pressed and retention of an adhesive bond after removal of the pressure [81]. The oligomeric polymer with low molecular weight provides tackiness, while the polymer with high molecular weight gives strength to the adhesive joint [45,82].

Asphaltenes were produced from Ashalcha heavy crude oil (Tataria, Russia). The fractional composition of the oil includes 23.1% saturated compounds, 45.6% aromatic compounds, 23.8% resins, and 7.5% heptane-insoluble asphaltenes, while its density and viscosity were 0.962 g·ml^–1^ and 4.25 Pa·s, respectively, at 20 °C [83,84]. Oil deasphalting was carried out with hexamethyldisiloxane as a precipitant at the volume precipitant/oil ratio of 15/1 according to the procedure described in detail earlier [59,85]. Asphaltenes do not have a distinct chemical structure [58]. They are a fraction of crude oil, consisting of hundreds of individual compounds. These compounds have in common the presence of one or more polyaromatic cores consisting of 3–7 benzene rings, while on the periphery of these cores, there are hydrocarbon substituents that may contain polar functional groups. The precipitated asphaltenes had a weight-average molecular mass of 828 g/mol, contained C 82.3 wt%, H 9.1 wt%, S 5.8 wt%, and N 1.6 wt%, and their detailed composition has been previously described [86].

The asphaltene/PIB compositions were prepared on a Polydrive twin-rotor mixer (Haake, Vreden, Germany) using sigma-blade rotors. Oppanol B 100 and Oppanol B 12 (1/2 wt/wt) were mixed at 180 °C with a gradual decrease in temperature to 120 °C for an hour. After that, Indopol H-1900 and the remaining part of Oppanol B 12 were added to the blend at 120 °C, and the mixing was performed at 10 rpm (about 8 s^−1^ [87]) for 6 h. In the end, a filler-free adhesive (PIB) was produced. The subsequent blending of PIB and asphaltenes was performed in the same mixer at 120 °C for 30 min at the same mixing speed. The mixtures containing 5, 10, 20, and 30 wt% of asphaltenes (*w*_asph_) were prepared.

The films for further testing were shaped on an HLCL-1000 laminator (ChemInstruments, Fairfield, OH, USA) between two layers of siliconized poly(ethylene terephthalate) film at 120 °C. The thickness of the formed adhesive films was 130 ± 20 μm.

### 2.2. Methods

The morphology of the adhesives was evaluated by the microphotographs obtained by optical microscopy using an objective with 100× magnification and a digital camera with a 12 MP 1/1.7″-type IMX226 CMOS image sensor (Sony, Tokyo, Japan).

The rheological properties were investigated using a rotation stress-controlled rheometer Discovery HR-2 (TA Instruments, New Castle, DE, USA) at 25 °C and 120 °C using a cone–plate unit (with a cone diameter of 25 mm, and an angle between the conical surface and the plate of 2°). Dependences of steady-state viscosity (*η*) on shear stress (*σ*) were obtained in the mode of stepwise increase in shear rate from 0.001 s^−1^ to 100 s^−1^. Amplitude dependences of the storage (*G*′) and loss (*G*″) moduli were measured at an angular frequency of 6.28 rad·s^−1^ with a variation of the strain amplitude from 0.01% to 200%, and the use of the first harmonic of the shear stress for calculations [88,89]. Frequency dependences of the same moduli were obtained in the region of linear viscoelasticity of samples with a strain amplitude of 0.1 % and a variation of the angular frequency in the range from 628 rad·s^−1^ to 0.628 rad·s^−1^. Temperature dependences of the storage and loss moduli were estimated at a strain amplitude of 0.1%, an angular frequency of 6.28 rad·s^−1^, and a temperature decrease rate of 3 °C/min. The equations for the calculation of the rheological parameters can be found elsewhere [90]; their standard deviations did not exceed 5%.

Adhesion tests were carried out using standard techniques and standard steel surfaces, as the bonding of glass plates with the adhesives and their subsequent lap-shear tests resulted in glass crumbling rather than breaking the adhesion bond. For this reason and the desire to assess the effect of asphaltenes on adhesive properties, the standard adhesion pull-off strength was evaluated using a TA.XT plus texture analyzer (Stable Microsystems, Godalming, UK) at 25 °C. A steel cylindrical rod (9.94 mm in diameter, surface roughness of 0.5 μm) was lowered to the surface of the sample film at a velocity of 0.1 mm/s and was pressed against the sample with a force of 0.5 kgf (which corresponds to a pressure of 16 kPa) for 10 s. In turn, the opposite surface of the sample film was pre-glued to a soda–lime glass plate. Then, the rod was pulled off at a rate of 0.1 mm s^−1^, and the resulting force was measured. In the process of destruction of the adhesive joint, there was either cohesive destruction of the sample film or its detachment from the steel rod, i.e., the adhesion to steel was less than to glass. The adhesive properties were assessed by the maximum pull-off force per adhesive surface area (by the apparent adhesion strength) and by the debonding energy of the adhesive joint (the area under the load–displacement curve [91]). The peel resistance at an angle of 90° was estimated using a TT-1100 tensile tester (ChemInstruments, Fairfield, OH, USA) at a peeling rate of 30.5 cm min^−1^ and a temperature of 25 °C. Before the test, an adhesive film of around 10-cm long and 12.5-mm thick was glued to a polished steel plate and rolled three times using a standard 2-kg roller. Based on the experiments, the mean peel force was calculated. For each formulation, at least five samples were tested.

The UV-Vis transmission spectra of the samples were measured using a UV 1900i spectrophotometer (Shimadzu, Kyoto, Japan) in a wavelength range from 800 nm to 200 nm. The samples were adhesive films (3-cm long, 15-mm wide, and 130-µm thick) glued to quartz glass plates, whereas a pure quartz glass plate was used as a reference sample.

## 3. Results and Discussion

### 3.1. Rheology of Adhesives in the Hot State

**Figure 1:**
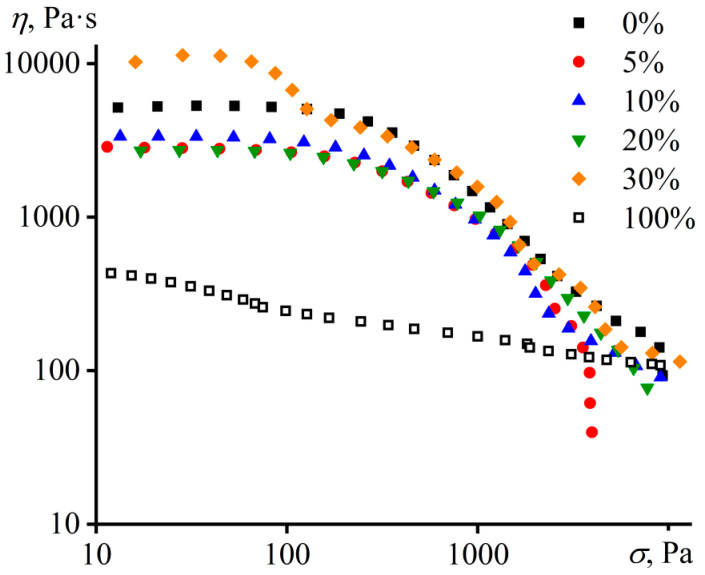
The dependences of viscosity on shear stress for PIB, asphaltenes, and their blends at 120 °C. The concentration of asphaltenes is shown in the legend.

The hot filler-free polymer matrix exhibits non-Newtonian behavior: the viscosity of PIB is constant in the region of low shear stresses but starts to decline at higher shear stresses because of a reduction in the density of macromolecular entanglements (Figure 1). Asphaltenes in the molten state have 10-times lower viscosity and less pronounced non-Newtonian behavior in comparison to PIB at the same temperature (Ostwald–de Waele flow behavior index of 0.855 versus 0.484 for PIB). As a result, the introduction of up to 20 wt% of asphaltenes into PIB leads to a decrease in its viscosity over the entire stress range, which can be explained by the partial solubility of lower-viscous asphaltenes in the polymer medium. With the rise in the asphaltene concentration up to 30 wt%, the low-shear viscosity of the mixture becomes higher than that of the original polymer matrix. This is probably due to the formation of droplets from insoluble asphaltenes, since the viscosity of the emulsion is usually higher than the viscosity of the continuous medium, even in the case of its polymer nature [92].

**Figure 2:**
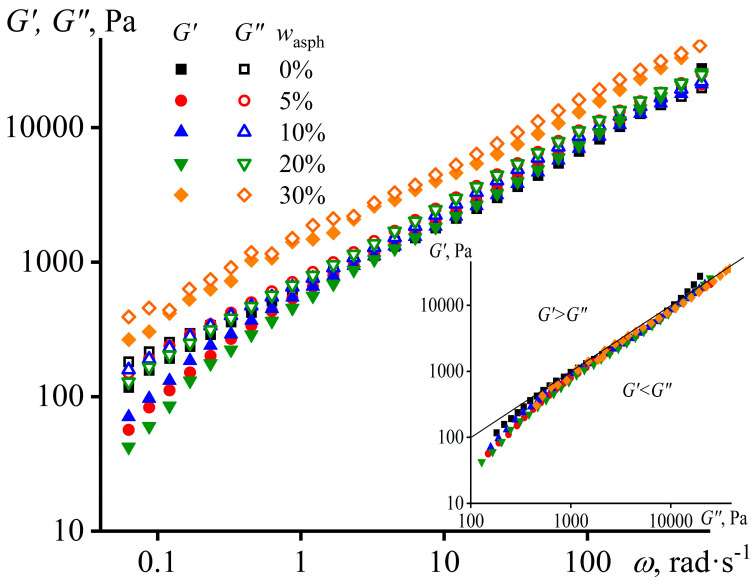
The frequency dependences of storage and loss moduli of PIB and its mixtures with different content of asphaltenes at 120 °C. The insert shows the same dependences but as Cole–Cole diagrams.

The additional way to evaluate the rheological and structural features of polymer mixtures is to study their viscoelasticity. Firstly, the measurement of frequency dependences of storage and loss moduli, which respectively characterize elasticity and internal friction of a material, allows for evaluating its behavior as solid- or liquid-like under different loading or observation conditions [60]. Secondly, it allows for an assessment of its structure because if a polymer melt exhibits solid-like behavior, it implies its heterogeneous and structured state. In our case, the storage and loss moduli of the hot filler-free adhesive have about the same values and monotonically decline with a decrease in the angular frequency (Figure 2), i.e., the sample demonstrates an intermediate behavior between the ones of a viscous liquid and an elastic solid over a wide range of observation times. This specific behavior is explained by the wide molecular weight distribution of PIB’s macromolecules and, accordingly, by the wide spectrum of their relaxation times, which is owing to the mixing of three polymers with very different molecular masses. The introduction of up to 20 wt% of asphaltenes results in the fact that the loss modulus slightly exceeds the storage modulus in the all frequency range, i.e., the asphaltenes make the behavior of the polymer more liquid-like. At the same time, the growth in the asphaltene concentration up to 30 wt% leads to an increase in both moduli, which may be due to the contribution of the viscoelasticity of the asphaltene/PIB interface to the overall viscoelasticity of the mixture [93,94]. Nevertheless, if we examine the dependences of the moduli on the Cole–Cole plot (the insert in Figure 2), then they overlap regardless of the asphaltene content. This means that the structure of all samples is very similar [95], i.e., asphaltenes do not fundamentally change the structure of hot PIB at 120 °C.

**Figure 3:**
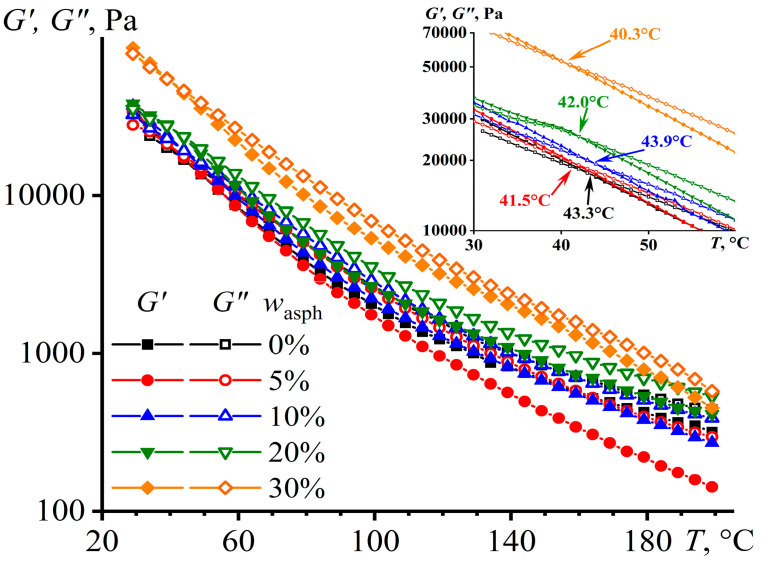
The temperature dependences of storage and loss moduli of PIB and its mixtures with different content of asphaltenes. The inset shows enlarged sections of the same curves, and the arrows indicate the points of equality of the storage and loss moduli.

The difference between the moduli increases upon heating above 120 °C, and the loss modulus starts to exceed the storage one (Figure 3), i.e., the samples behave more liquid-like. When cooled, both moduli rise, but the storage modulus increases more intensively and starts to exceed the loss modulus at a certain temperature (see the inset in Figure 3). The point of equality of the moduli can be considered as the nominal liquid–solid transition temperature [96]. Since the dominance of elastic behavior in comparison to flow behavior is important for an adhesive after its application (to suppress its cold flow behavior and make it creep-resistant), this temperature can be used to evaluate the thermal stability of the adhesives. In our case, asphaltenes slightly reduce thermal stability, but within 3 °C. This may be because a soluble part of asphaltenes acts as a plasticizer, which reduces the adhesive’s viscosity and thermal stability.

### 3.2. Rheology of Adhesives at Normal Temperature

**Figure 4:**
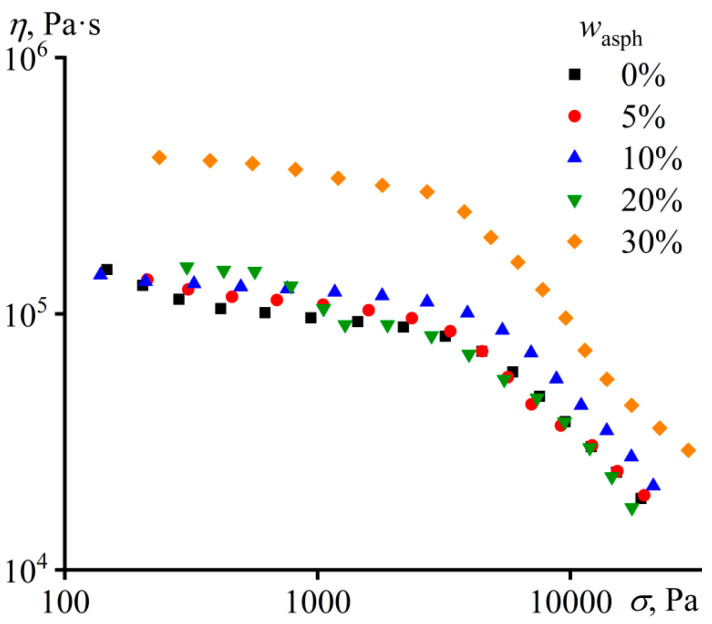
The dependences of viscosity on shear stress for PIB and its mixtures containing a different mass fraction of asphaltenes at 25 °C.

The plasticizing effect of asphaltenes disappears when the adhesives are cooled to 25 °C: the viscosity of mixtures containing asphaltenes is comparable to or even exceeds the viscosity of pure PIB (Figure 4). In the case of 30 wt% of asphaltenes, the viscosity of the mixture turns out to be four times higher than the viscosity of PIB, whereas their viscosities differed only two times at 120 °C (see Figure 1). Thus, as the temperature decreases, asphaltenes lose their solubility in PIB and start to act as solid particles. In turn, this leads to a change in the viscoelastic properties of the adhesives, but only for high content of asphaltenes.

**Figure 5:**
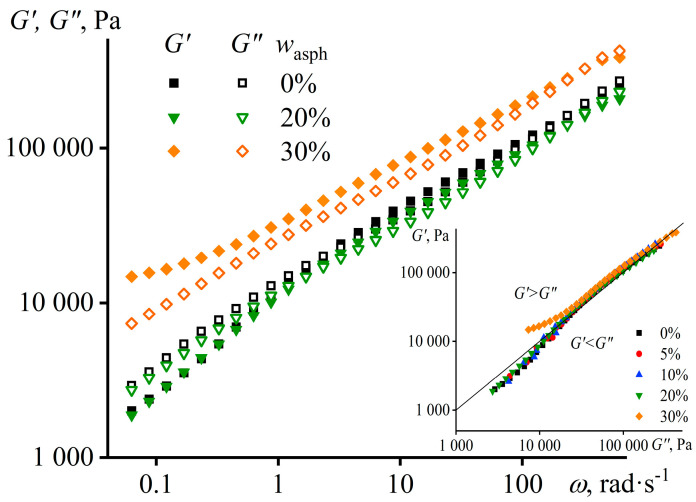
The dependences of storage and loss moduli on angular frequency for PIB and its mixtures containing a different mass fraction of asphaltenes at 25 °C. The insert shows the same dependences but as Cole–Cole diagrams.

In the case of the introduction of up to 20 wt% of asphaltenes, the frequency dependences of the storage and loss moduli at normal temperature practically coincide with those for pure PIB (Figure 5). The loss modulus exceeds the storage modulus in the low-frequency region, meaning that the mixtures behave like liquids, and this allows them to fill the irregularities of the glued surfaces and form adhesive joints. The storage modulus exceeds the loss one at higher frequencies; i.e., the mixtures behave predominantly solid-like, retaining the formed adhesive joint in case of an attempt to quickly destroy it (within 0.01–1 s corresponding to an angular frequency of 1–100 rad·s^–1^). The transition from liquid-like to solid-like behavior with increasing frequency of external action is typical for polymer melts and is described by the Maxwell model. It is a transition from the terminal zone (where *G*′ < *G*″, *G*′~*ω*^2^, and *G*″~*ω*) to the rubbery plateau (where *G*′ > *G*″ and *G*′ ≈ *const*), occurring when the angular frequency is inversely proportional to the relaxation time of macromolecular chains. According to the reptation theory, the relaxation time is proportional to the cube of the polymer’s molecular weight. For this reason, this transition is deeply masked in our case due to the very wide molecular weight distribution of the adhesive’s macromolecules and the resultant presence of numerous relaxation times (their extremely broad spectrum [97]). The behavior of systems with many relaxation times is described by a set of many Maxwell elements connected in parallel with each other [61]. As a result, the rubbery plateau and the terminal zone degenerate due to multiple overlaps, and the transition between them becomes almost indistinguishable. In this case, the adhesives’ stickiness results from physical interactions consisting of filling in the irregularities of the glued surfaces and weak van der Waals interactions with them instead of chemical interactions with the formation of covalent bonds with these surfaces. It means that all the adhesives are potentially removable rather than permanent. However, removal of an adhesive can occur with or without its residue on the bonded surfaces; i.e., with respectively cohesive or adhesive failure of the adhesive joint. In our case, the cohesion fracture would occur if the detachment of the adhesives is at low velocity when *G*″ > *G*′ and they behave like liquids. In contrast, the adhesive fracture requires high detachment rates when *G*″ < *G*′ and the adhesives behave like elastic bodies. Meanwhile, in the latter case, there is a cohesive fracture of the glass itself when gluing and then fast detaching the two glass plates, while adhesive fracture can happen by gluing either two steel surfaces or glass and steel ones.

The addition of 30 wt% of asphaltenes results in the fact that the storage modulus is higher than the loss modulus in the all-considered frequency range, takes higher values, and exceeds the loss modulus more strongly at low frequencies. Moreover, the introduction of asphaltenes causes a change in the structure of the adhesive in this case, since its dependence *G*′(*G*″) on the Cole–Cole diagram does not coincide with the dependences of other samples that overlap with each other (the insert in Figure 5). Note that there was no such discrepancy at 120 °C (see the insert in Figure 2), meaning that a decrease in temperature caused a deterioration in the compatibility of asphaltenes and the polymer matrix, which, however, is evident only at a 30% mass fraction of asphaltenes. In this respect, the results of tests at 120 °C can be considered a reference point for assessing the structural and rheological changes in the compositions after cooling. The discrepancy is observed for points obtained at low frequencies when the storage modulus tends to reach a plateau. The constancy of the storage modulus at low frequencies, when it exceeds the loss modulus (i.e., there is solid-like behavior), may indicate the formation of a structural network from particles [98,99].

**Figure 6:**
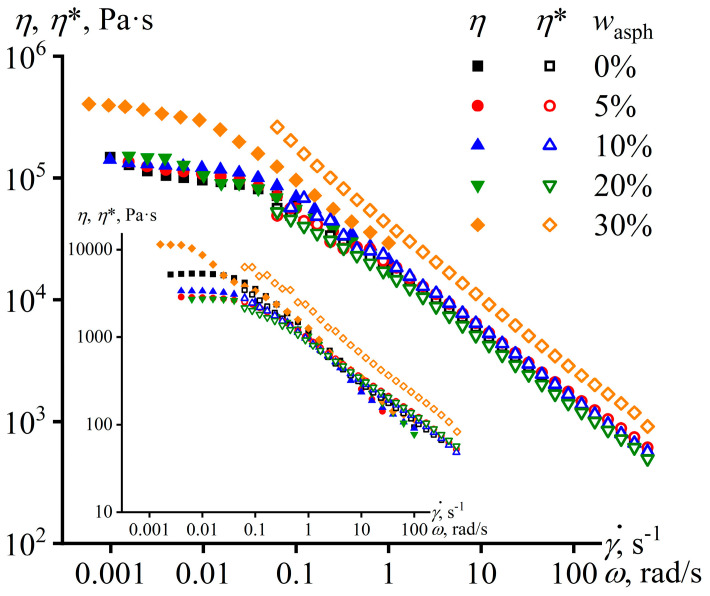
The dependences of steady-state viscosity and complex viscosity on, respectively, shear rate and angular frequency for PIB and its mixtures containing a different mass fraction of asphaltenes at 25 °C (the mainline figure) and 120 °C (the insert).

The formation of the structural network is indirectly confirmed when comparing the steady-state viscosity with the complex one (Figure 6). According to the Cox-Mertz rule, these viscosities coincide when the shear rate and angular frequency are numerically equal. This rule works well for regular polymer melts and solutions whose viscoelasticity results from macromolecular entanglements, but it is inaccurate when the viscoelasticity is wholly or partially due to colloidal effects. In our case, the rule is satisfied for PIB containing up to 20% asphaltenes, but it fails for the asphaltenes’ mass fraction of 30% at both 25 °C (the mainline part of Figure 6) and 120 °C (the insert to Figure 6). The latter means that the adhesive is heterogeneous at both of these temperatures and its disperse phase contributes to viscoelasticity. At 25 °C, this contribution is due to the viscoelasticity of the structural network from asphaltene particles. In turn, at 120 °C, it can be associated with the viscoelasticity of molten asphaltene droplets.

**Figure 7:**
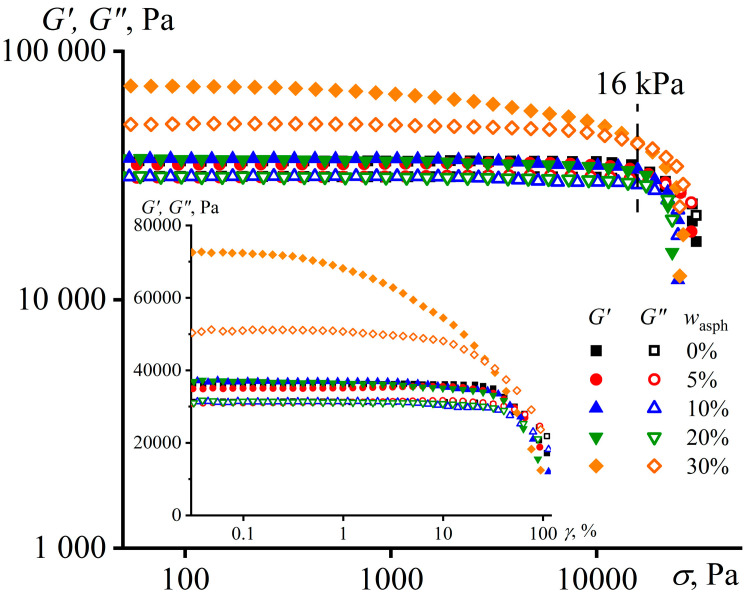
The dependences of storage and loss moduli on shear stress for PIB and its mixtures with different content of asphaltenes at an angular frequency of 6.28 rad/s and 25 °C. The insert shows the same results but as a function of strain.

On the one hand, this transformation of viscoelastic properties at 30% asphaltene mass fraction should contribute to a significant increase in the apparent strength of adhesive joints, as the formation of a structural network increases the cohesive strength of the adhesive and suppresses the cohesive fracture of its joints. On the other hand, the same structural network may prevent the formation of adhesive joints, as the storage modulus exceeds the loss modulus even at low frequencies, meaning that the material might behave as an elastic body, which is incapable of irreversible (plastic) deformations for filling the irregularities of the gluing surfaces, and hence, of being tacky. Nevertheless, the adhesive joints are formed in our case. This is due to the destruction of the structural network of asphaltene particles due to the action of pressure applied to the adhesive. The structural network is destroyed under high stress, the material ceases to behave as an elastic body, and instead exhibits the properties of a liquid for a brief moment until the external load is removed and the structural network is restored, meaning that the structural network has a coagulation nature [100]. This suggestion is proved by the amplitude dependences of the storage and loss moduli obtained for pure and asphaltene-containing PIBs (Figure 7).

For pure PIB and PIB containing less than 30 wt% of asphaltenes, the region of linear viscoelasticity (constancy of *G*′ and *G*″) extends up to a strain amplitude of about 20% (see the insert in Figure 7). The deformation of samples with a larger amplitude leads to a decrease in the moduli due to the partial stretching of macromolecules and a change in the number of entanglements between them. At the same time, a decline in the storage and loss moduli for the mixture containing 30 wt% of asphaltenes starts at a strain amplitude as low as around 0.4%. This may indicate the presence in its volume of a rather fragile structural network of asphaltene particles, which is already destroyed at small strains. However, the storage modulus markedly declines with an increase in strain, while the loss modulus remains almost constant. Thus, the network of asphaltene particles strongly increases the elastic properties of the mixture, but has little effect on the energy dissipation caused by internal friction. When the strain amplitude reaches about 10%, the loss modulus starts to noticeably decrease, probably due to the onset of changes in the conformations of macromolecules. Nevertheless, the storage modulus decreases more rapidly with increasing strain amplitude than the loss modulus in all cases. Even if a sample exhibits a solid-like behavior at small strains, then it becomes liquid-like at large strains.

In our case, pull-off tests were carried out by applying a stress of about 16 kPa to the mixtures (see experimental part). At this shear stress, the storage modulus of the specimen containing 30 wt% of asphaltenes is comparable in magnitude to its loss modulus (Figure 7). In this respect, even the most filled adhesive can exhibit stickiness and fill the roughness of the bonded surfaces under these loading conditions, like other samples with a lower asphaltene content.

### 3.3. Adhesion Characteristics of Adhesives

**Figure 8:**
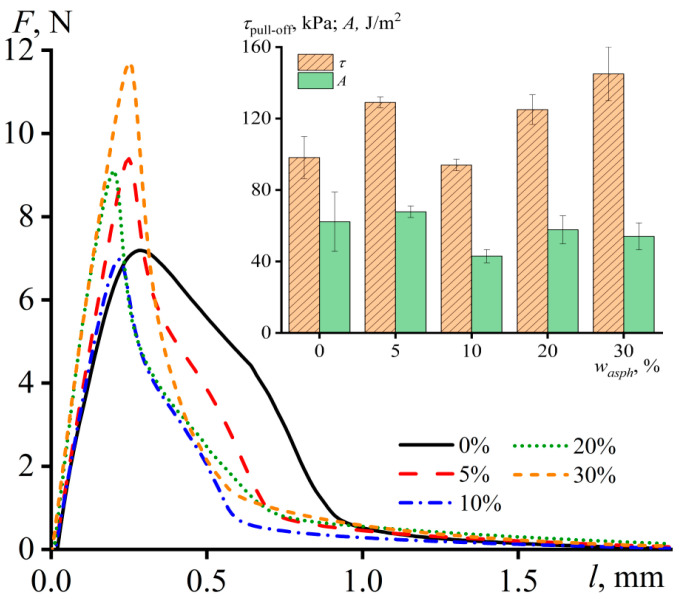
Load–displacement curves for probe-tack tests of PIB and its mixtures containing a different mass fraction of asphaltenes at 25 °C. The insert demonstrates the dependences of the debonding energy and the apparent strength of adhesive joints on the asphaltene content.

The results of measuring the force arising in the detachment process of adhesives from the substrate are shown in Figure 8. The curve for the unfilled PIB has an asymmetric form with a shoulder in the region of large displacements. This shoulder indicates the cohesive nature of the failure of adhesive bonds and is due to the formation of fibrils from the adhesive and their gradual stretching. The addition of asphaltenes causes a decrease in the shoulder area, contributing to the suppression of both the adhesive flexibility and the cohesive nature of the destruction of its bonds. Moreover, the asphaltene content of 30 wt% causes a noticeable increase in the apparent strength of adhesive bonds, which is defined as the maximum recorded force at their destruction (*τ*_pull-off_, the insert in Figure 8). In addition, the area under the load–displacement curve makes it possible to estimate the debonding energy (*A*), i.e., the apparent work of adhesive joint destruction, which is approximately the same regardless of the asphaltene content in the adhesive. Since the cohesive shoulder on the load–displacement curves disappears, the invariance of the apparent work of adhesion is associated with a gradual transition from the cohesive nature of the adhesive bond destruction to the adhesive one under the influence of asphaltenes.

**Figure 9:**
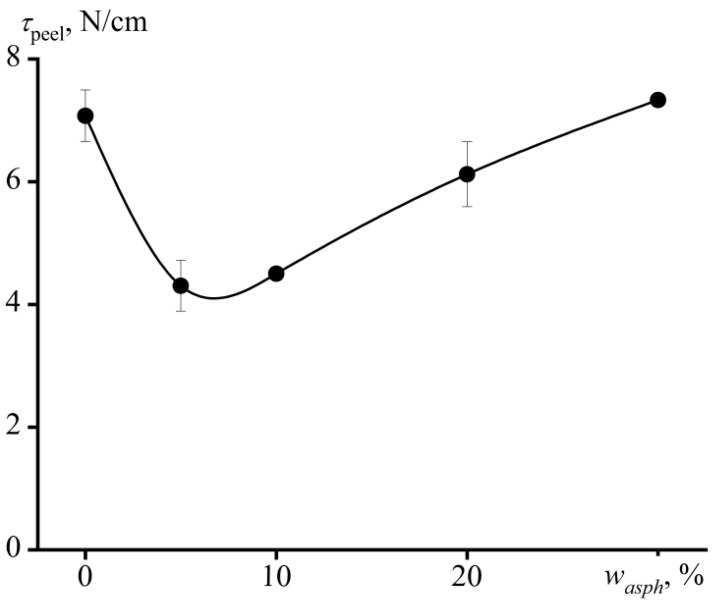
The dependence of peel strength on the asphaltene content in adhesives.

In the case of peeling of adhesives from the surface at an angle of 90°, the destruction of adhesive joints has an adhesive nature for all samples. However, the addition of up to 20 wt% of asphaltenes causes a significant drop in the apparent adhesive strength (*τ*_peel_, Figure 9). Furthermore, the nature of the detachment acquires a peel–elongation mode, when the detachment occurs in jerks with periodic stretching of the adhesive layer, detachment of its part, new stretching, detachment, and so on, all over again. Thus, the decrease in the peel strength can be associated with the irregular impact-induced manner of the detachment, leading to a periodic sharp increase in local stresses and a reduction in the overall apparent adhesive strength. The introduction of 30 wt% of asphaltenes suppresses the flexibility of the adhesive, making the manner of destruction of the adhesive bonds uniform and leading to an increase in their apparent strength to the initial level that is typical for unfilled PIB.

Therefore, asphaltenes at a mass fraction of 5–10% act as tackifiers for the polymer matrix, probably because they are partially soluble in the matrix and act similarly to oligomeric compounds used for the same purpose [25,26,27,28,45,101]. Since the polymer matrix initially has a good tackiness, its additional plastification harms the apparent strength of the adhesive joints, likely due to a decrease in cohesion strength. However, the apparent strength of adhesive joints increases at a content of asphaltenes greater than 10% when they start to play the role of a filler reinforcing the polymer matrix [41,42,43,44,45,46,47,50,54]. The role of asphaltenes as solid particles increases to a maximum when their mass fraction reaches 30% and they form a structural network that effectively reinforces the adhesive, eventually causing the transition from cohesive to adhesive fractures of its adhesive joints. In other words, asphaltenes exhibit dualistic behavior, being both plasticizing and strengthening agents, depending on the concentration, which has been previously shown for other polymer matrices [66,75,77,78].

### 3.4. Optical Properties of Adhesives

**Figure 10:**
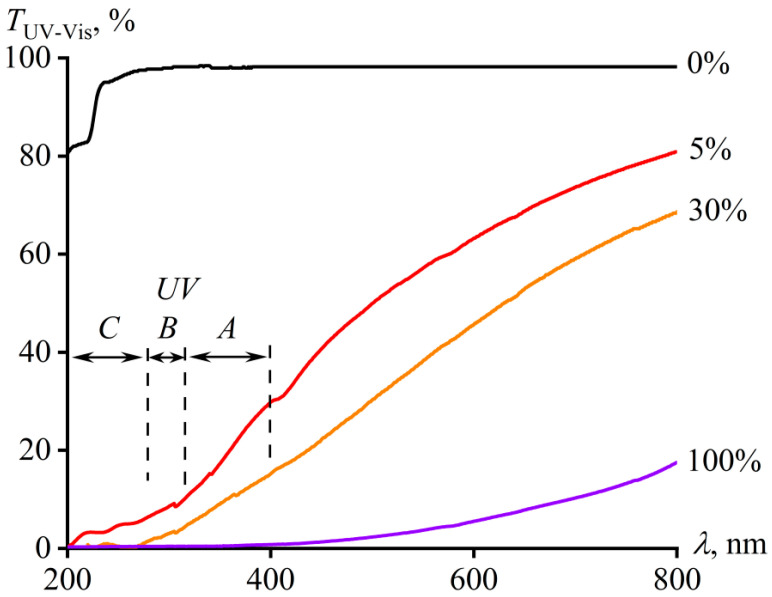
The dependence of the transmittance on the wavelength of electromagnetic radiation for PIB, asphaltenes, and their mixtures. The mass fraction of asphaltenes is indicated at the curves.

Pure PIB is optically transparent at wavelengths greater than 290 nm (Figure 10). In the case of shorter-wave radiation, unfilled PIB absorbs it, but only to a limited extent, reaching about 20% at a polymer film thickness of 130 μm. In contrast, pure asphaltenes are almost completely non-transparent at wavelengths below 400 nm, although their transmittance starts to gradually rise as the wavelength increases above this value. Thus, pure asphaltenes effectively absorb all three types of solar UV radiation (UV-A, -B, -C; ranges are shown in Figure 10). At the same time, asphaltenes are partially transparent in the visible light region, which makes it possible to use them for creating semi-transparent sticky films through their introduction into PIB. Indeed, according to the spectra of adhesive films containing asphaltenes, they are semi-transparent in the entire considered region of electromagnetic radiation. Asphaltene-filled adhesives partially transmit UV radiation, but much less in comparison to the transmission of visible light. In this case, the more the adhesive contains asphaltenes, the higher its absorption in the entire spectral region. As a result, the addition of 30 wt% of asphaltenes causes complete non-transparency of the PIB-based adhesive in the UV-C region, as well as greatly reduced UV-A and UV-B transmission.

**Figure 11:**
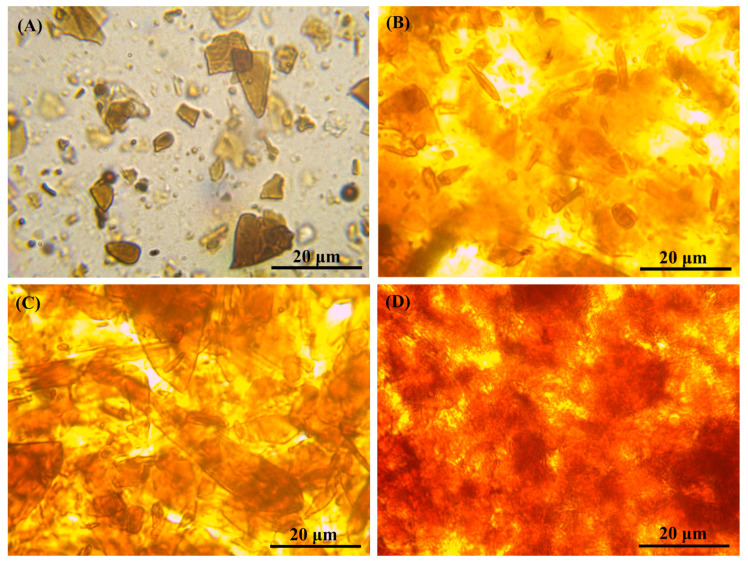
Microphotographs of PIB containing 5 (**A**), 10 (**B**), 20 (**C**), or 30 (**D**) wt% of asphaltenes.

If we consider the morphology of the mixture containing 5 wt% of asphaltenes (Figure 11A), then asphaltenes can be detected as flat brown glassy fragments in a transparent polymer medium. It can be assumed that the light passing through the filled adhesive partially goes through these glassy fragments with the absorption of UV radiation along with a part of the visible light. Since there are places in the adhesive that do not contain asphaltenes, a part of the radiation appears to pass through it without changing the spectral characteristics. This causes incomplete absorption of UV radiation by the adhesive. The introduction of 10 wt% of asphaltenes results in their filling of the entire visible space of the polymer matrix with a change in its color to yellow-brown (Figure 11B). An asphaltene content about of 20–30 wt% causes the overlapping of asphaltene fragments on each other in the considered adhesive layer (Figure 11C), with a color change up to red due to the multiple superimposing of numerous asphaltene fragments in the case of the mixture containing 30 wt% of asphaltenes (Figure 11D).

## 4. Conclusions

Asphaltenes are semi-transparent microsized glassy splinters that can improve both the UV-absorbing and operating characteristics of polyisobutylene pressure-sensitive adhesives. Partial absorption of visible light by asphaltenes leads to a brown-red color of the adhesive in a thin layer, which does not allow for obtaining colorless glass plates glued with it. However, this feature can be used to create tinted UV-protective car windows, eyeglass lenses, windowpanes for museums and office buildings, and other glass sheets for similar applications requiring UV absorption. The use of a 30% asphaltene mass fraction is the most optimal, improves the adhesive characteristics of the pressure-sensitive adhesive, and gives it the highest ability to absorb UV radiation. The application field of asphaltenes as low-cost UV absorbers is novel, gives them additional value, and opens new ways for their rational disposal in obtaining useful products.

## Data Availability

The data presented in this study are available on request from the corresponding author.
